# Smartwatch Versus Routine Tremor Documentation: Descriptive Comparison

**DOI:** 10.2196/51249

**Published:** 2024-03-20

**Authors:** Catharina Marie van Alen, Alexander Brenner, Tobias Warnecke, Julian Varghese

**Affiliations:** 1 Institute of Medical Informatics University of Münster Münster Germany; 2 Department of Neurology and Neurorehabilitation Klinikum Osnabrück – Academic Teaching Hospital of the University of Münster Osnabrück Germany

**Keywords:** Parkinson disease, tremor, smart wearables, smartwatch, mobile apps, movement disorders, tremor documentation, tremor occurrence, tremor score

## Abstract

We addressed the limitations of subjective clinical tremor assessment by comparing routine neurological evaluation with a Tremor Occurrence Score derived from smartwatch sensor data, among 142 participants with Parkinson disease and 77 healthy controls. Our findings highlight the potential of smartwatches for automated tremor detection as a valuable addition to conventional assessments, applicable in both clinical and home settings.

## Introduction

Clinical assessment of tremor is limited by its subjectivity, making it challenging to detect subtle tremors (<0.05 g) [1]. The need for technology-based evaluations led to extensive research on sensor-based tremor assessments [2-6]. However, the benefits of smart consumer devices for clinical documentation remain unaddressed. We aimed to bridge this research gap by comparing tremor documentation from routine neurological assessments with a Tremor Occurrence Score (TOS) derived from smartwatch sensor recordings. We included 142 participants with Parkinson disease (PD) and 77 healthy controls. During clinical visits, routine neurological assessments were conducted and wrist-motion data were captured using a previously established Smart Device System (SDS) in a controlled setting [1,7]. The SDS is based on consumer smartwatches and smartphones. The smartwatches were validated in a geophysical experiment for tremor analysis with comparison to a gold-standard seismometer [1]. We investigated the potential of complementing routine tremor documentation with tremor capture using smartwatches [8].

## Methods

### Overview

The SDS parent study [1] recruited 450 participants, including patients with PD and other movement disorders and healthy controls. The SDS implemented data capture through electronic questionnaires and app-guided movement tasks with smartwatch sensing. Varghese et al [7] provide a detailed description of the study. Of the 450 participants, 101 were excluded due to diagnoses other than PD. Moreover, 130 patients with PD without explicit information regarding tremor in routine documentation were excluded, resulting in 219 participants. Table 1 summarizes the age, sex, and years from diagnosis onset distribution.

**Table 1:** Overview of sex, age, and years to diagnosis onset for the 2 study cohorts.

Characteristics				PD^a^ cohort (n=142)		HC^b^ cohort (n=77)		*P* value	
**Sex, n (%)**									<.001
	Female			42 (29.6)		48 (62.3)			
	Male			100 (70.4)		29 (37.7)			
Age (y), mean (SD)				64.9 (9.4)		62.6 (12.4)		.08	
**Years from diagnosis onset**									N/A^c^
	Mean (SD)			6.70 (5.36)		N/A			
	**Range, n (%)**								
		0-4	60 (42.2)		N/A				
		5-9	39 (27.5)		N/A				
		10-14	31 (21.8)		N/A				
		15-19	6 (4.2)		N/A				
		≥20	6 (4.2)		N/A				

^a^PD: Parkinson disease.

^b^HC: heathy control.

^c^N/A: not applicable.

Patients with PD were labeled as either (1) *TremorDocumented*: tremor was documented in routine documentation, regardless of type or amplitude (78/142, 54.9%), or (2) *NoTremorDocumented*: no information regarding tremor in routine documentation (64/142, 45.1%). We compared the routine tremor documentation to a Tremor Occurrence Score (TOS) from smartwatch recordings of a 20-second task, during which participants remained seated in an armchair. The TOS is normalized, from 0=no tremor occurrence to 1=tremor occurrence with a clear frequency peak, with 0.5 representing the average TOS of healthy controls. Details on calculations can be found in Multimedia Appendix 1 [9,10].

### Ethical Considerations

The SDS parent study has been registered (ClinicalTrials.gov NCT03638479) and approved by the ethical board of the University of Münster and the physician’s chamber of Westphalia-Lippe (reference 2018-328-f-S). All participants gave written informed consent, and data were pseudonymized.

## Results

**Figure 1:**
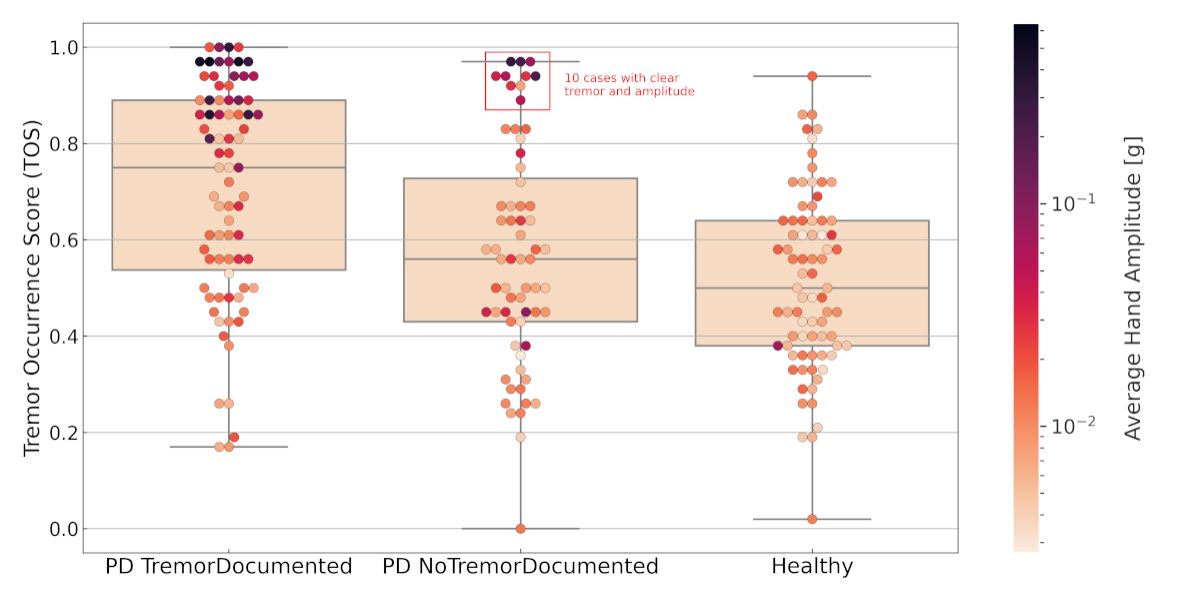
Boxplots of Tremor Occurrence Scores (TOSs) for all participants grouped by their routine tremor documentation. Each data point represents a participant of the respective group. Points are colored according to the average wrist amplitude in grams. The middle line is the median. The top and bottom sections are upper and lower quartiles, respectively. The box represents the middle 50% of TOSs for the group. The upper and lower whiskers represent scores outside the middle 50%. PD: Parkinson disease.

Figure 1 shows boxplots of the TOSs according to participants’ routine tremor documentation. Overall, 16 (25%) out of 64 patients in the *NoTremorDocumented* group had a TOS ≥0.75 (the median TOS in the *TremorDocumented* group) and an average amplitude of 0.07 g. Particularly, 10 (16%) patients in the *NoTremorDocumented* group had a clear tremor (TOS >0.88, amplitude >0.10 g). The *TremorDocumented* group had a TOS ≥0.75 and an average amplitude of 0.11 g. Overall, patients with a clear but low-amplitude tremor (TOS ≥0.75, amplitude <0.05 g) were 1.59 times less likely to have a documented tremor (*NoTremorDocumented*) than those with a nonsubtle tremor (TOS ≥0.75, amplitude >0.05 g).

## Discussion

Smartwatches can capture clear tremors in certain patients with PD, wherein the tremor was not documented during routine neurological assessments. Generally, patients with a clear but low-amplitude tremor (<0.05 g) were 1.59 times less likely to have a documented tremor than those with a strong tremor. We hypothesize that the tremor may not have been documented during the examination because of its small amplitude and difficulty of being seen. Moreover, a few patients (10/64, 16%) in the *NoTremorDocumented* group showed a clear tremor with visible amplitudes (>0.10 g), possibly due to the tremor being unnoticed or absent during routine examination. Likewise, tremors may not always manifest during smartwatch-based data capture, ultimately affecting the findings’ reproducibility.

Nevertheless, we suggest that integrating smartwatch-based data capture into routine neurological assessments enhances tremor documentation by providing objective numbers on frequency and amplitude for biomarker analysis. Smartwatch integration would further enable improved longitudinal assessment at home. The findings are not suitable for assessing the potential of diagnostic improvements, as routine manual documentation was only compared to smartwatch-based data capture. The parent study is currently evaluating a larger patient cohort, including differential diagnosis, to evaluate the diagnostic accuracy by using machine learning and more detailed tremor features, other movement characteristics, and nonmotor symptoms [1].

## Appendix

Multimedia Appendix 1 Tremor Occurrence Score (TOS) and tremor characteristic calculation.

## Abbreviations

PD: Parkinson disease
SDS: Smart Device System
TOS: Tremor Occurrence Score
