# Supplementary material for: Smartwatch Versus Routine Tremor Documentation: Descriptive Comparison
Source: JMIR Form Res. 2024 Mar 20;8:e51249. doi: 10.2196/51249 (PMC10993114; doi:10.2196/51249)
Supplement: Multimedia Appendix 1 [file formative_v8i1e51249_app1.docx]

# Multimedia Appendix 1

## Methods

### Tremor Occurrence Score (TOS)

For tremor occurrence score (TOS) calculations, we used acceleration and angular velocity sensor recordings from a resting task, in which the participant rested in an armchair for 20 seconds. The task was selected because of its lack of interference with voluntary or repetitive movement and ease of reproducibility in home-based assessments. All sensor orientations x,y,z, as well as both wrists, were used, yielding 12 time series channels per participant (2 sensors * 3 axes * 2 wrists). The smartwatches recorded at a sample frequency of 100 Hz for 20 seconds. The first 50 samples were removed from each channel to eliminate signal content from the smartwatch vibration that signaled the beginning of a recording. Each channel was band-pass-filtered from 3.5 to 12 Hz to remove low-frequency components, including drift, DC offset, and involuntary movements, as well as to exclude high-frequency components outside the scope of interest in tremor analysis. [9,10]

Given the oscillatory nature of tremor, the score was calculated in the frequency domain. For each channel, successive Fourier Transforms were computed using Hanning windows comprising 256 samples, with a 50 % overlap. The TOS was calculated for each Fourier Transform by taking the ratio of the peak power to the total spectral power. Here, the peak power is defined as the sum of the spectral power within a frequency range of +-1 Hz centered around the dominant frequency. The dominant frequency was estimated by identifying the frequency that corresponds to the highest power value in the tremor frequency range of 3.5 Hz to 12 Hz. The total spectral power was defined as the power estimation across the entire spectrum from 0.1 Hz to 20 Hz. Subsequently, the median value of the three highest ratios of peak power to the total spectral power was calculated to compute a TOS for the respective channel. The TOS was calculated for all 12 channels and the highest TOS per participant was used for analysis. The TOS was normalized such that it ranges from 0 (no tremor occurrence at all) to 1 (tremor occurrence with clear frequency peak), and a score of 0.5 as a reference that represents the average TOS of HC.

On the sensor axis with the highest tremor score, we calculated the average wrist amplitude in units of g for each participant. The channel was divided into 10 equal-length sections, and for each section, the peak-to-peak amplitude was computed. Then, the average of all 10 peak-to-peak amplitudes was calculated.
